# Microbial mechanisms to transform the super-trace element tellurium: a systematic review and discussion of nanoparticulate phases

**DOI:** 10.1007/s11274-023-03704-2

**Published:** 2023-07-29

**Authors:** Yuru Wei, Sihan Yu, Qian Guo, Owen P. Missen, Xian Xia

**Affiliations:** 1 Hubei Key Laboratory of Edible Wild Plants Conservation & Utilization, Hubei Engineering Research Center of Characteristic Wild Vegetable Breeding and Comprehensive Utilization Technology, Huangshi Key Laboratory of Lake Environmental Protection and Sustainable Utilization of Resources, Hubei Normal University, Huangshi, P. R. China; 2 Centre for Ore Deposit and Earth Sciences, University of Tasmania, TAS, Private Bag 79, Hobart, 7001 Australia

**Keywords:** Tellurium, Toxicity, Resistance, Microbial transformation, Tellurium nanoparticles (TeNPs)

## Abstract

Tellurium is a super-trace metalloid on Earth. Owing to its excellent physical and chemical properties, it is used in industries such as metallurgy and manufacturing, particularly of semiconductors and – more recently – solar panels. As the global demand for tellurium rises, environmental issues surrounding tellurium have recently aroused concern due to its high toxicity. The amount of tellurium released to the environment is increasing, and microorganisms play an important role in the biogeochemical cycling of environmental tellurium. This review focuses on novel developments on tellurium transformations driven by microbes and includes the following sections: (1) history and applications of tellurium; (2) toxicity of tellurium; (3) microbial detoxification mechanisms against soluble tellurium anions including uptake, efflux and methods of reduction, and reduced ability to cope with oxidation stress or repair damaged DNA; and (4) the characteristics and applications of tellurium nanoparticles (TeNPs) produced by microbes. This review raises the awareness of microorganisms in tellurium biogeochemical cycling and the growing applications for microbial tellurium nanoparticles.

## History and application of Tellurium

### Discovery and usage

Tellurium (Te) was first discovered in 1783 from the tellurium-bearing gold mining area of the Metaliferi Mountains, in modern-day Romania (Emsley 2011). Its crustal abundance is low, averaging around 0.005 mg/kg (Wedepohl 1995). It had few early uses, though it was typically found in gold-bearing areas, most notably in the Kalgoorlie gold mining area in Western Australia where gold-bearing tellurides contain ~ 25% of the total gold endowment of this world-class system (Vielreicher et al. 2016). Tellurium is a metalloid element which, when alloyed, endows different properties to different metals. The microbiological response to tellurium was first described over a century ago, with the now-characteristic blackening brought about by the formation of tellurium nanoparticles observed by King and Davis (1914). Despite many suggestions of the potential utility of tellurium for (micro)biological applications, the role of tellurium in microbiology and pharmacology remains poorly studied (Presentato et al. 2019; Hosseini et al. 2023).

**Fig. 1:**
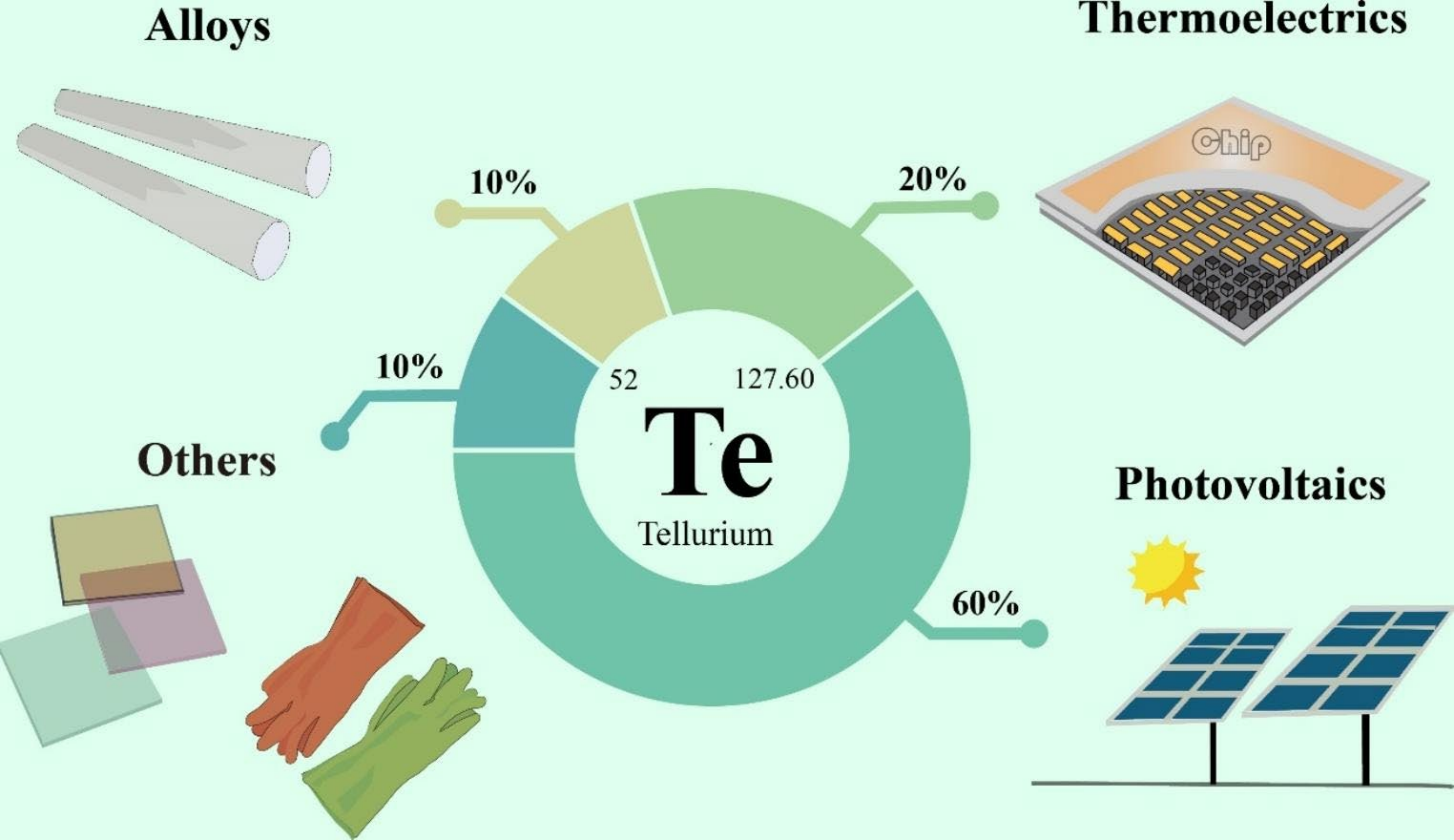
The applications of tellurium

The usage of tellurium may have also increased dramatically if tellurium compounds had been used to prevent engine-knocking of combustion engines – despite their efficacy, their smell meant that insidious tetraethyl lead was used instead, to environmentally detrimental effect (Midgley Jr 1937). Today, the usage of tellurium is primarily as tellurides in solar panels (cadmium telluride) (60%) and thermoelectric devices (bismuth telluride) (20%) (Nassar et al. 2022). Other uses include in alloys of a range of metals (with steel and copper to improve machinability, with lead to improve vibration resistance) (10%), in the processing of rubber, and as a pigment in glasses and ceramics (10%)(Nuss 2019; Anderson 2022) (Fig. 1).

### Growing demand and criticality

The importance of tellurium as a commodity is increasing, primarily by virtue of the growing demand for tellurium in cadmium telluride solar panels. Cadmium telluride solar panels make up 5–10% of the solar panel market share. Despite this growing global usage of tellurium (at least 580 tonnes production in 2022, up from ~ 140 tonnes two decades ago) (Anderson 2022), only a handful of sites refine tellurium directly as a commodity, including Kankberg, Sweden (producing gold and tellurium) and more recently, the Kennecott Mine, Utah, USA (producing copper, gold, silver, molybdenum and now tellurium). Tellurium is most readily recovered from the anode slimes produced by the electrowinning of copper (Makuei and Senanayake 2018), but a move towards heap leaching for copper recovery may mean that tellurium is no longer produced from copper mining. Tellurium is often classed as a Critical Metal, particularly in the United States of America (McNulty and Jowitt 2021), where it is produced from just two refineries. The fact that Te is listed on criticality lists at all is testament to the importance of having efficient refinement pathways for by-product metal(loid)s. More than 10 times the global tellurium supply is mined every year, with the majority reporting to tailings dams and waste rock storage facilities. Mine waste storage has a high monetary cost to mine operators and potentially an environmental cost if deleterious elements leach from storage facilities (Kavlak et al. 2013; Missen et al. 2020).

### Environmental issues

**Fig. 2:**
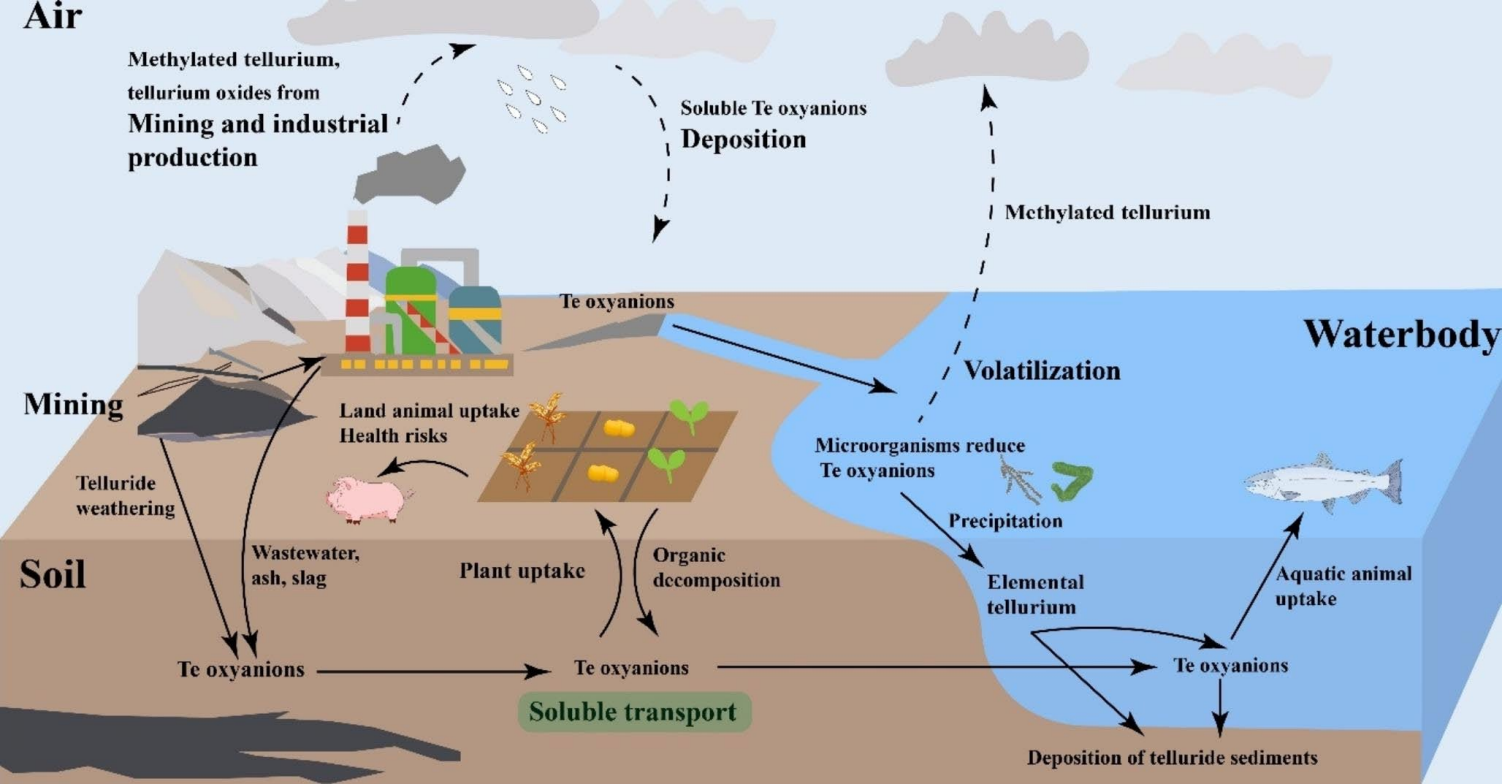
The cycle of tellurium on the earth’s surface

Despite its high toxicity to both microscopic and macroscopic life, tellurium’s rarity has meant that it has not left a negative environmental legacy in the manner of elements such as lead and arsenic. Tellurium is concentrated unequally in the environment, with high levels of Te in certain gold and copper deposits despite its overall low crustal abundance (Grundler et al. 2013), including extreme examples of enrichment above 0.1 wt% in ores (e.g. Börner et al. 2021) believed to be related to its mobility in (boiling) hydrothermal fluids (Cooke and McPhail 2001). Tellurium is usually present in aqueous environments in trace concentrations less than 1 µg/L (Llaver et al. 2021). Tellurium is most often one of a suite of contaminant elements present in settings such as acid mine drainage of Te-bearing pyrites (Zhan et al. 2022). However, the few studies that do exist suggest that tens of thousands of tonnes of tellurium have been released to the environment during industrial activities which process Te-rich feedstocks, with 9500 tonnes estimated to have been released to the atmosphere from copper smelters alone (Wiklund et al. 2018). Due to the extensive occurrence of tellurium and its oxyanions in various industrial and metal mining activities, tellurium has recently caused environmental pollution concerns (Alavi et al. 2020). After being released into the environment, wastewater containing tellurium ions may accumulate in soil and aquatic systems (Qin et al. 2017; Curtin et al. 2020) (Fig. 2). Tellurium is found primarily in the form of oxyanions, tellurite (TeO_3_^2−^) and tellurate (TeO_4_^2−^), in natural waters and weathered surface environment geological samples (Wu et al. 2014; Grygoyc and Jablonska-Czapla 2021). Soluble tellurite is more toxic than tellurate, and elemental tellurium (Te^0^) is less toxic than both (Yao et al. 2021). The distribution of soluble tellurium needs to be monitored in industrial settings such as copper-processing facilities.

### Toxicity of Tellurium

#### General toxicity

As well as impacting plant growth and poisoning microorganisms, exposure to Te can also affect the health of humans and animals. Animal experiments have shown that exposure to tellurite accelerates liver toxicity and oxidative stress in rat liver tissues (Safhi et al. 2016). Cases of non-occupational tellurium exposure were rarely reported due to most natural settings and consumer products containing little significant tellurium. However, some groups have reported that tellurium in the soil might lead to contamination of foods. Tellurium concentrations in foods are generally < 1 mg Te / kg food and total human intake of Te per day is likely no more than 0.1 mg (Gerhardsson 2022), although it is worth noting that these concentrations nonetheless represent a Te enrichment 1–2 orders of magnitude compared to the average Te concentration in soil (average ~ 0.027 mg/kg; Ba et al. 2010), with typical values such as fresh fruits (0.185 mg/kg), cereals (0.168 mg/kg), legumes (0.382 mg/kg), potatoes (0.189 mg/kg), meat (0.686 mg/kg), nuts (1.072 mg/kg), fishes (0.803 mg/kg) and some dairy products (0.937 mg/kg) (Filippini et al. 2020; Gad and Pham 2014). As little 2 mg/kg tellurium in drinking water can pose a threat to human health (Yao et al. 2022). The amount of Te in the human body is not well-studied, but it is likely to be less than 1 mg (Emsley 2011).

Once tellurium compounds enter cells, they could induce cellular reactions including (1) interference of thiols (redox enzymes); (2) replacement of Se and S in proteins; (3) damage of cell membrane structure; and (4) increased oxidative stress (Goff et al. 2021a; Tang et al. 2022; Reddy et al. 2023). Among them, the production of reactive oxygen species (ROS) induced by tellurium oxyanions is the main factor of tellurate toxicity (Peng et al. 2022). The metabolism of tellurium in the human body remains unclear, despite being first recognized as a potential industrial poison a century ago (Shie and Deeds 1920). Recently, Duan et al. (2023) indicated a significant correlation between tellurium exposure and an increased risk of developing hypertension (high blood pressure). Animal studies showed that up to 25% of tellurium dioxide (TeO_2_) taken orally was absorbed by the gut; tellurium was primarily stored in the kidneys, but it accumulates in the liver, spleen, heart, lungs, brain, and bones (Hayes and Ramos 2019) (Fig. 2). Accidental ingestion of tellurium-containing metal-oxidising solutions by two children (independent of each other) led to symptoms including vomiting, difficulty swallowing, blackened tongue and lips and a garlic odour to the breath (Yarema and Curry 2005). To our knowledge, only two deaths have been recorded from tellurium ingestion due to accidental poisoning by sodium tellurite at a very high concentration of 30 mg sodium tellurite per kilogram body weight (Keall et al. 1946; Gerhardsson 2022). Symptoms included vomiting, kidney pain, loss of consciousness and a strong garlic odour. Systemic effects of acute tellurium toxicity in rats include lethargy, gastrointestinal disease, and fur changes, while chronic toxicity includes peripheral neuropathy, cerebral cortex changes, kidney and liver lesions, and reproductive effects (Gerhardsson 2022). The factors of toxicity, such as the generation of reactive oxygen species (ROS), disruption of cell membrane structure, and interference with redox enzymes, which occur in microbial cells, are also applicable in the toxicological assessment of animal cells (Safhi et al. 2018). Currently, there is a lack of research on the toxicological mechanisms of tellurium, particularly in humans and animals, compared to other group 16 counterpart, selenium, which has been more extensively studied (in part due to being a biologically essential element).

### Microbial toxicity

Tellurite is toxic to both prokaryotes and eukaryotes. Tellurium compounds may act as antibacterial agents, effectively inhibiting the growth of infectious microorganisms such as *Escherichia coli*, *Salmonella typhi* and *Klebsiella pneumoniae*, and could be used to treat diseases such as syphilis, tuberculosis and leprosy (Goff 2020; Vavrova et al. 2021). In addition, ammonium trichloro tellurate (an organotellurium compound) also provided an option for the treatment of some symptoms caused by HIV infection (Peng and Li 2019).

Tellurite anions are toxic to most microorganisms at concentrations as low as 1 µg/ml (Arenas-Salinas et al. 2016). These soluble tellurium anions are highly toxic to most bacteria and more toxic than metals such as mercury and lead (Harrison et al. 2004; Workentine et al. 2008). Tellurite anions in high concentrations could alter balance in soil microbial communities. Kolesnikov (2019) found that the tellurium contaminated (even at 0.003 mg/kg) chernozem (black, humus-rich) soil showed a decrease in total bacterial count, reduced abundance of *Azotobacter*, and no significant recovery trend in the soil’s biological characteristics for 90 days after contamination. Among the soil pollution caused by silver, bismuth, tellurium and thallium, tellurium and thallium were the most ecotoxic, based on their effects on soil enzyme (catalase and dehydrogenase) activity, soil bacterial count and wheat root length (Kolesnikov et al. 2022). These studies demonstrated the high toxicity of tellurium and its significant harm to soil ecology. As tellurite anions exhibit toxicity to bacteria at low aqueous concentrations (1 µg/ml), pore water in tellurium-rich tailings contains high concentrations of salts and potentially toxic elements that might affect ectopic microbial communities (Hayes and Ramos 2019), selecting for tellurium-resistant microorganisms as a result. In addition, tellurite and tellurate extracted from semiconductor materials showed toxicity to the marine bacterium *Aliivibrio fischeri* (Ramos-Ruiz et al. 2016b).

#### Microbial detoxification mechanisms for tellurium

**Fig. 3:**
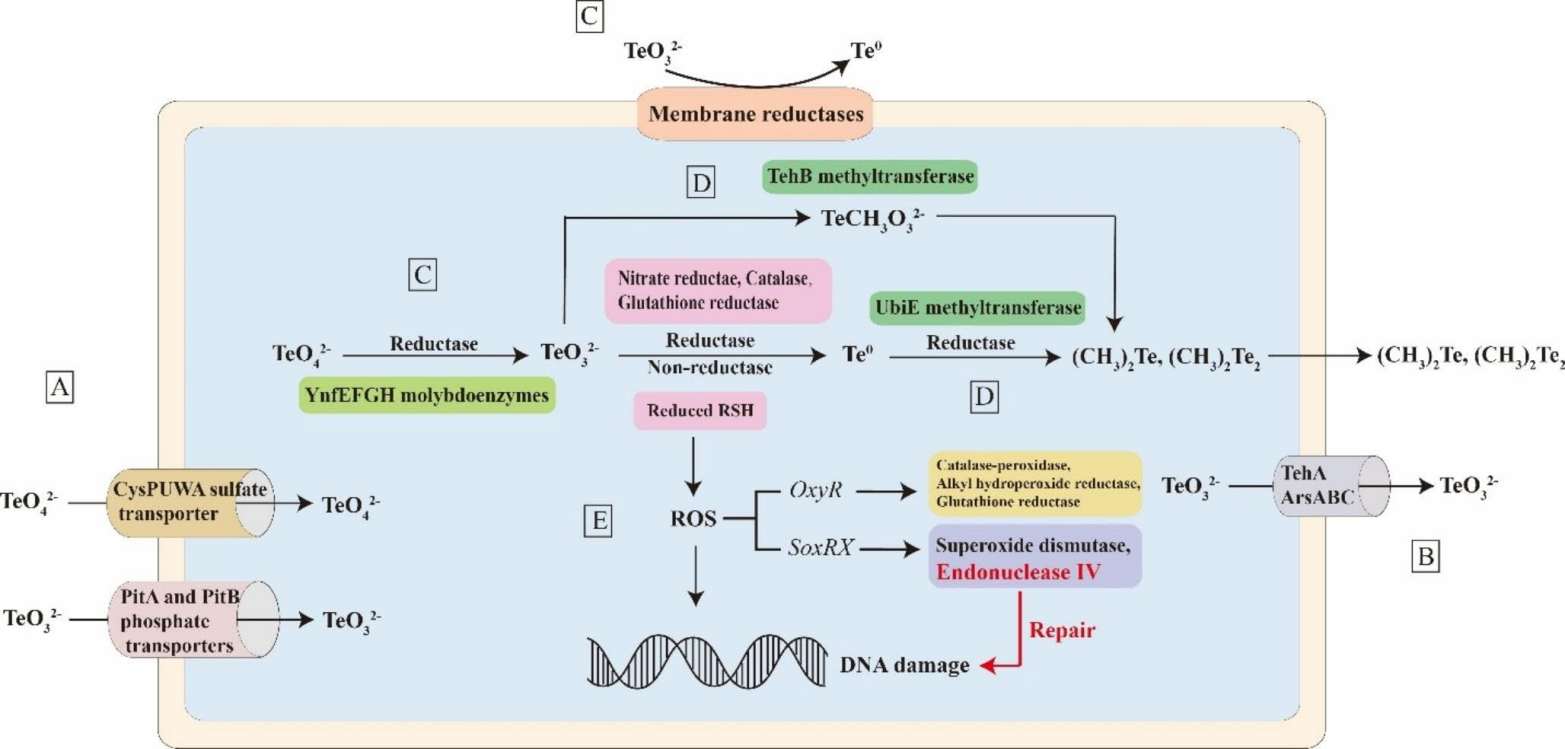
The microbial detoxification mechanism to tellurite and tellurate. (**A**) reduce uptake of tellurite, (**B**) efflux of tellurite, (**C**) reduction of tellurite and tellurate to insoluble elemental tellurium [Te(0)], (**D**) reduction of tellurite and tellurate to methylated tellurium [Te(-II)], (**E**) reducing oxidative stress and DNA damage repair

Various tellurium oxyanion detoxification mechanisms have been identified in microorganisms. The main detoxification mechanisms is reduction by either precipitation or methylation, and others include decreased intake, efflux, and reducing oxidative stress (Fig. 3).

#### Transport of tellurate

Transport is the first step in tellurium oxyanion metabolism in cells. As there are no known specific tellurite or tellurate transferases, tellurium oxyanions must enter cells via other anionic transferases. Goff and Yee (2017) discovered that in *E*. *coli* K-12, deletion of the CysPUWA sulfate transporter permease or ATPase subunits prevents tellurate entry into the cytoplasm, thus conferring higher resistance to tellurate.

#### Reduction of tellurate

The primary tellurium detoxification mechanism for many microorganisms is the transformation of tellurite or tellurate to elemental tellurium through biological reduction (Wu et al. 2019). In *E. coli* K-12, molybdopterin-containing enzymes were found to be capable of reducing tellurate to elemental tellurium (Theisen et al. 2013). A membrane-associated tellurite reductase isolated from the *Erythromonas ursincola* strain KR99 is also able to reduce tellurate to elemental tellurium (Maltman et al. 2017a). In addition, cysteine could also reduce tellurate to elemental tellurium in *E*. *coli* K-12 (Goff et al. 2021a).

Baesman et al. (2007) speculated that *Sulfurospirillum barnesii* cells might use a two-step reduction pathway to reduce tellurate, first reducing it to tellurite, and then further reducing tellurite to elemental tellurium. Ramos-Ruiz et al. (2016a) found that the reduction rate of tellurite was seven times faster than that of tellurate in a methanogenic microbial consortium. Since the reduction of tellurate requires more electrons to convert them to elemental tellurium compared to tellurite (6 per Te cation rather than 4), it takes longer to transfer the additional electrons (Maltman et al. 2017a). Therefore, tellurite accumulates relatively less during the reduction of tellurate in a community of mixed microorganisms in anaerobic, methane-producing granular sludge (Ramos-Ruiz et al. 2016a).

### Transport of tellurite

Microorganisms can enhance their resistance to tellurite by reducing their tellurite intake. It is known that in the tellurite metabolism and transport system, the phosphate transporters PitA and PitB are responsible for the uptake of tellurite (Montenegro et al. 2021). Similarly, acetate transport proteins could take up tellurite in some bacteria, and the resistance of these bacteria to tellurite could increase when the competitor acetate was present (Borghese et al. 2010). In a *Micromonospora* strain isolated from a metal-rich environment, increasing the saturation and branched chain fatty acids may stiffen the cell membrane, resist excessive tellurium oxyanions entering the cell membrane, and avoid oxidative bursts associated with tellurite in order to cope with high concentrations (5 mM) of tellurite (Piacenza et al. 2022). Additionally, surface adsorption of tellurite could also control tellurite uptake (Goff et al. 2021b). These findings suggested that the reduced intake may contribute to tellurium oxyanion detoxification.

Efflux of tellurite out of cells is also a detoxification mechanism used by some microorganisms. In these bacteria, the tellurite resistant operon TehA encodes an intimal protein and TehB encodes a methyltransferase (Choudhury et al. 2011). *E. coli* TehA (EcTehA) is a typical efflux protein that could eliminate tellurite out of cells (Choudhury et al. 2011). In *Aeromonas hydrophila*, cytoplasmic protein TehB may accelerate the rate of reduction of tellurite to elemental tellurium (Castro et al. 2020). Additionally, the arsenite efflux system ArsABC had been reported to export tellurite (Turner et al. 1992).

### Reduction of tellurite

#### Reduction of tellurite to element tellurium

Microorganisms convert tellurite into elemental tellurium or volatile methylated forms of tellurium through reduction as a detoxification mechanism (Ollivier et al. 2011). Microbially mediated reduction by precipitation (from soluble tellurium oxyanions to insoluble elemental tellurium) can be divided into enzymatic or non-enzymatic reduction. In terms of non-enzymatic reduction, some reducing thiols, such as glutathione, can also directly reduce tellurite to elemental tellurium (Muñoz-Diaz et al. 2022). Various organic electron shuttles (such as lawsone, menadione, anthraquinone-2-sulfonate, and anthraquinone-2,6-disulfonate) were also reported to mediate tellurite reduction (Wang et al. 2011; Borghese et al. 2020). Additionally, Fe^3+^ could promote electron generation and electron transfer to accelerate tellurite reduction in *Shewanella oneidensis* MR‑1 (Kim et al. 2013; He et al. 2021).

Numerous tellurite reductases have now been identified in microbes (Table 1). The first confirmed tellurite reductase was discovered in *Mycobacterium avium* (Castro et al. 2008). Subsequently, the tellurite reduction ability of nitrate reductase in *E*. *coli*, *Ralstonia eutropha*, *Paracoccus denitrificans*, and *Paracoccus pantotrophus* has been confirmed by both in vivo and in vitro experiments in many bacteria (Borghese et al. 2017). Calderon et al. (2006) found that the enzyme catalase in animals and *Staphylococcus epidermidis* could also act as a tellurite reductase. Castro et al. (2008) and Miguel et al. (2010) found that the dihydrolipoamide dehydrogenase in *Aeromonas caviae* ST, *E. coli*, *Zymomonas mobilis*, *Streptococcus pneumoniae*, and *Geobacillus stearothermophilus* exhibited good tellurite reduction activity. This indicated that dihydrolipoamide dehydrogenase may also be a common tellurite reductase. Glutathione reductase in *Pseudomonas* sp. BNF22 (Pugin et al. 2014), and 6-phosphogluconate dehydrogenase in *E. coli* (Sandoval et al. 2015) also showed good tellurite reduction activity. Additionally, a 117 kDa membrane protein in *E. ursincola* KR99 (Maltman et al. 2017a), TrxR in *Bacillus* sp. Y3 (Yasir et al. 2020), Mycothione reductase in *Rhodococcus erythropolis* PR4 (Butz et al. 2020) and Flagellin (FlaA) in *Paenibacillus pabuli* ALJ109b (Farias et al. 2021) were also proven to be acting as microbial tellurite reductases.

**Table 1:** Microbial Te (IV) reductase

Reductase	Cell localization	Measurement condition	Michaelis constant Km (mM)	Maximum reaction rate Vmax (U/mg)	Source	Ref.
Nitrate reductase	Cell membrane /periplasmic	Purified protein	0.6	0.97	*E. coli*, *R. eutropha*, *P. denitrificans*, *P. pantotrophus*	(Sabaty et al. 2001)
Catalase	Unknown	Cell extract /Partially purified proteins	0.9	Unknown	*S. epidermidis*	(Calderon et al. 2006)
Dihydrolipoamide dehydrogenase (E3)	Unknown	Purified protein	0.04794	84.5	* A. caviae* ST, *E. coli, Z. mobilis, S. pneumonia, G. stearothermophilus*	(Castro et al. 2008; Arenas-Salinas et al. 2016)
Glutathione reductase (GorA)	Cytoplasm	Purified protein	0.08947	6314	*Pseudomonas* sp. BNF22, *E. coli*	(Pugin et al. 2014; Arenas-Salinas et al. 2016)
6-phosphogluconate dehydrogenase (6PGD)	Unknown	Purified protein	Unknown	Unknown	*E. coli*	(Sandoval et al. 2015)
Thioredoxin reductase (TrxB)	Unknown	Purified protein	0.1145	9586	*Staphylococcus haemolyticus* BNF01, *E. coli*	(Arenas-Salinas et al. 2016)
Alkyl hydroperoxide reductase (AhpF)			0.8196	77,875		
NADH: flavorubredoxin reductase (NorW)			0.6949	5347		
Putative oxidoreductase (YkgC)			0.5171	2696		
Mercuric reductase (MerA)			Unknown	Unknown		
Membrane reductase	Cell membrane	Purified protein	3.36	5.15	*E. ursincola* KR99	(Maltman et al. 2017a)
Periplasmic reductase	Periplasmic	Purified protein	3.9	5.6	*Shewanella frigidimarina ER-Te-48*	(Maltman et al. 2017b)
Thioredoxin-disulfide reductase (TrxR)	Unknown	Purified protein	16.31	12.23	*Bacillus* sp. Y3	(Yasir et al. 2020)
Mycothione reductase (Mtr)	Unknown	Purified protein	0.779 ± 0.050	Unknown	*R. erythropolis* PR4	(Butz et al. 2020)
Flagellin (FlaA)	Flagellum	Purified protein	Unknown	Unknown	*P. pabuli* ALJ109b	(Farias et al. 2021)

Interestingly, Arenas-Salinas et al. (2016) found that most reported reductases for tellurite were flavin adenine dinucleotide (FAD) based flavin reducing proteins according to bioinformatics analysis. Therefore, they expressed and purified flavoproteins including thioredoxin reductase (TrxB), alkylhydroperoxide reductase (AhpF), glutathione reductase (GorA), mercuric reductase (MerA), NADH: flavorubredoxin reductase (NorW), dihydrolipoamide dehydrogenase (E3), and inferred oxidoreductase YkgC from *E. coli* and other environmental strains (Arenas-Salinas et al. 2016). The in vitro enzyme activity tests showed that these proteins could reduce tellurite to generate tellurium nanoparticles (Arenas-Salinas et al. 2016).

Currently, studies on tellurite reductases are mainly focused on aerobic conditions. The reduction mechanism of tellurite under anaerobic conditions is not very clear. A diverse community of metal(loid) oxide respiring bacteria around black smokers is known to remove tellurium (and other metalloid) oxyanions as terminal electron acceptors through anaerobic respiration (Maltman et al. 2016). However, this electron transport pathway needs further study.

### Reduction of tellurite to methylated tellurium

Methylation is another method by which microbes detoxify tellurium by reduction. Primarily, tellurite methylation proceeds by conversion to dimethyl telluride [(CH_3_)_2_Te, DMTe], which is less toxic than tellurite, highly volatile and has a characteristic garlic odour (Prigent-Combaret et al. 2012). Other methylated tellurium species produced include dimethyl ditelluride [(CH_3_)_2_Te_2_, DMDTe] and the mixed species dimethyltellurenyl sulfide [(CH_3_)_2_TeS, DMTeS] (Ollivier et al. 2008). The most commonly identified methylation pathway was S-adenosylmethionine-dependent methylation (Choudhury 2013). DMTe has been identified as a product of tellurium bioreduction in many bacterial strains, such as *Scopulariopsis brevicaulis* and *Pseudomonas fluorescens* (Choudhury 2013). In a *Penicillium* strain, DMTe was not produced when only tellurium was present, but DMTe could be detected when selenium and tellurium were added together (Choudhury 2013). This suggested that the presence of selenium might induce tellurium methylation pathways.

Tellurite methylation is usually catalyzed by methyltransferase. Overexpression of the bacterial thiopurine methyltransferase (bTPMT) in *E. coli* can enhance its resistance to tellurite (Choudhury et al. 2011). Currently, the most well-studied tellurium methyltransferase is TehB in *E. coli*, which is typically arranged together with TehA (Choudhury et al. 2011). TehB could convert tellurite to TeCH_3_O_3_^2−^, which further reacts to form DMTe (Chasteen et al. 2009; Choudhury 2013), in a process which does not produce elemental tellurium. Furthermore, Ollivier et al. (2011) found that aeration plays an important role in controlling the volatile and precipitation equilibrium of tellurite oxyanions, where elemental tellurium may be an intermediate in volatilization pathways in tellurite resistant *Rhodotorula mucilaginosa*. In some bacteria tellurite can be first reduced to elemental tellurium, which is then methylated by the UbiE methyltransferase to generate DMTe (Araya et al. 2004). Similarly, in the tellurite-reducing bacterium *Sporosarcina* sp. Te-1, the formed elemental tellurium can further be transformed into methylated organotellurium compounds (Wang et al. 2021).

### Reduced oxidative stress and DNA damage repair

Tellurite or tellurate react with reduced thiols or reductases after entering the cell, leading to production of reactive oxygen species (ROS) (Calderon et al. 2006; Diaz-Vasquez et al. 2014). The production of ROS might further lead to DNA damage and affect the normal growth and reproduction of microorganisms. ROS production is the main reason why Te oxyanions are so toxic to many microbes.

Microbes can upregulate some genes including genes which promote (1) reduction of ROS or (2) repair DNA damage caused by ROS to cope with the increasing ROS. For instance, the production of ROS led to the up-regulation of global regulatory factors such as OxyR and SoxRX in *E. coli*; *OxyR* further regulated proteins such as catalase-peroxidase (KatG), alkyl hydroperoxide reductase (AhpCF), glutathione reductase (GorA), glutaredoxin A (GrxA), and thioredoxin C (TrxC) to cope with oxidative stress (Chasteen et al. 2009; Pérez et al. 2007; Zannoni et al. 2008). *SoxRS*, on the other hand, up-regulated proteins such as superoxide dismutase (SodA) and endonuclease IV to deal with the toxicity of ROS produced by tellurite (Chasteen et al. 2009; Choudhury 2013). KatG, AhpCF, GorA, TrxC and SodA could reduce ROS content in intracellular environments, meanwhile endonuclease IV could repair DNA damage.

### Characterization and application of tellurium nanoparticles synthesized by microorganisms

#### Biological tellurium nanoparticles (Bio-TeNPs) based on microbes

Numerous studies reported that soluble and highly toxic tellurium oxyanions could be converted into low toxicity elemental tellurium nanoparticles through the action of bacteria, fungi or archaea (Ao et al. 2022; Srivastava et al. 2015), which is not just a laboratory phenomenon, but also detectable in nature (Missen et al. 2022). Bio-TeNPs were produced from tellurite by microorganisms in most studies, and only a few bacteria have been reported to produce Bio-TeNPs from tellurate, such as *S. barnesii* and *Bacillus selenitireducens* (Baesman et al. 2007). This might be because reduction of tellurite to elemental tellurium occurs more readily than reduction of tellurate to elemental tellurium in microorganisms (Maltman et al. 2017a). This phenomenon was similar to the trends observed for selenium oxyanion metabolism in microorganisms (Wang et al. 2022).

Literature reports of tellurium oxyanion bioreduction are dominated by bacteria (Table 2), followed by fungi and archaea (Table 3). However, fungi and archaea are more resistant to high concentrations of tellurite than bacteria (Pearion and Jablonski 1999; Hosseini et al. 2023; Wu et al. 2019; Ao et al. 2022). Higher maximum tellurite resistance might be owing to the higher metal absorption capacity, bioaccumulation ability, effective extracellular enzyme secretion and/or the filamentous structure of fungi (Barabadi et al. 2018; Kashyap et al. 2018), and archaea exhibited high levels of resistance in extreme environments with high salinity, and had the ability to resist and reduce the toxic tellurite (Srivastava et al. 2015). However, most bacteria are sensitive to tellurite at significantly lower concentrations, with even 1 µM tellurite enough to cause a toxic response. Although bacteria are tolerant to lower concentrations of tellurite than fungi and archaea, they might be a good cell factory for the production of Bio-TeNPs for further applications. This is mainly due to the fact that bacteria possess some advantages such as (1) faster growth than fungi and archaea, (2) the fermentation technology is currently more mature, and (3) produced Bio-TeNPs are easier to extract from cultures. On the other hand, fungi and archaea suitable for large-scale fermentation and rapid reduction to form Bio-TeNPs remain to be further discovered.

**Table 2:** Bio-TeNPs produced by bacteria

Biological source	Location	Percursor	Shape	Size (nm)	References
*Acinetobacter pittii* D120	Unknown	Te(IV)	Rod-shaped	60–130	(Tang et al. 2022)
*Aromatoleum* sp. CIB	Intracellular	Te(IV)	Rod-shaped	200	(Alonso-Fernandes et al. 2023)
*B. selenitireducens*	Intracellular	Te(VI), Te(IV)	Rod-shaped, rosettes	200, 1000	(Baesman et al. 2007)
*Bacillus* sp. BZ	Cell debris	Te(IV)	Rod-shaped	20 × 180	(Zare et al. 2012)
*B. Selenitireducens*	Cell surfaces	Te(IV)	Rod-shaped, Rosettes	Unknown	(Wang et al. 2019)
*Escherichia*	Intracellular and extracellular	Te(IV)	Ellipse-shaped	0.9 × 10^− 3^-1.8 × 10^− 3^	(Nguyen et al. 2019)
*Lactobacillus plantarum* PTCC1058	Intracellular	Te(IV)	Spheres	45.7	(Mirjani et al. 2015)
*Lysinibacillus* sp. ZYM-1	Cell membrane	Te(IV)	Rod-shaped, hexagonal Te nanoplates, nanoflowers, and nanobranches	300–500	(Wang et al. 2018)
*Lysinibacillus* sp. EBL303	Intracellular	Te(IV)	Spheres	22–148	(Hosseini et al. 2023)
*Ochrobactrum* sp. MPV1	Intracellular	Te(IV)	Short needle-like	Unknown	(Zonaro et al. 2017)
*Ochrobactrum* sp. MPV1	Unknown	Te(IV)	Spherical	76.2	(Zonaro et al. 2015)
*Pseudomonas* sp. strain BNF22	Cytoplasm	Te(IV)	Round, porous	60	(Pugin et al. 2014)
*P. pseudoalcaligenes*	Cell debris	Te(IV)	Rod-shaped	185	(Forootanfar et al. 2015)
*P. pabuli* ALJ109b	Unknown	Te(IV)	Spheres	<100	(Mirjani et al. 2015)
*P. pseudoalcaligenes*	Unknown	Te(IV)	Individual, rod-shaped, rosettes	50–200	(Shakibaie et al. 2017)
*R. aetherivorans* BCP1	Intracellular	Te(IV)	Rod-shaped	148 ± 104, 223 ± 116	(Presentato et al. 2016)
*Rhodobacter capsulatus*	Extracellular	Te(IV)	Acicular	200–700	(Borghese et al. 2017)
*R. aetherivorans* BCP1	Cytoplasm	Te(IV)	Spherical, rod-shaped	> 700	(Presentato et al. 2018)
*Raoultella*	Intracellular and extracellular	Te(IV)	Rod-shaped	1.7 × 10^− 3^-2.6 × 10^− 3^	(Nguyen et al. 2019)
*R. capsulatus*	Extracellular	Te(IV)	Elongated needle-like	10 ± 5	(Borghese et al. 2020)
*S. barnesii*	Intracellular and extracellular	Te(VI), Te(IV)	Irregularly shaped spheres	< 50	(Baesman et al. 2007)
*Shewanella* baltica	Intracellular	Te(IV)	Rod-shaped	8–75	(Vaigankar et al. 2018)
*Shewanella* sp. NT-1	Intracellular	Te(IV)	Spheres	Unknown	(Sakaguchi et al. 2019)
*Shinella* sp. WSJ-2	Intracellular	Te(IV)	Rod-shaped	50–120	(Wu et al. 2019)
*Streptomyces cyaneus*	Extracellular	Te(IV)	Spherical TeO_2_ Nps	35–89	(El-Sayyad et al. 2020)
*Streptomyces graminisoli*	Unknown	Te(IV)	Rods, rosette	21.4	(Abed et al. 2023)

**Table 3:** Bio-TeNPs produced by fungi and archaea

	Biological source	Source	Percursor	Shape	Size (nm)	Ref.
Fungi	*Aspergillus welwitschiae*	Fungal isolate	Te(IV)	Elliptic to spherical	60.8	(Abo Elsoud et al. 2018)
	*Aureobasidium pullulans*	Supernatants	Te(IV)	Granular	40–70	(Liang et al. 2019)
	*A. pullulans*	Supernatants	Te(IV)	Spherical	5–65	(Nwoko et al. 2021)
	*A. niger*	Hyphae	Te(IV)	Rod-shaped, spheres	200–300, 20–100	(Sinharoy and Lens 2022)
	*Mortierella humilis*	Supernatants	Te(IV)	Granular	40–70	(Liang et al. 2019)
	*Mortierella* sp. AB1	Intracellular	Te(IV)	Rod-shaped	100–500	(Ao et al. 2022)
	*Phanero chaetechrysosporium*	Hyphae	Te(IV)	Needle-like	20–465	(Espinosa-Ortiz et al. 2017)
	*Penicillium chrysogenum* PTCC5031	Extracellular	Te(IV)	Spherical	33.8	(Barabadi et al. 2018)
	*Phoma glomerata*	Supernatants	Te(IV)	Pillar and needle shapes	40–70	(Liang et al. 2019)
	*P. glomerata*	Supernatants	Te(IV)	Needle-shaped	10–80	(Liang et al. 2020)
	*Trichoderma harzianum*	Supernatants	Te(IV)	Pillar and needle shapes	40–70	(Liang et al. 2019)
Archaea	*Halococcus salifodinae* BK3	Intracellular	Te(IV)	Hexagonal needle-shaped	10 × 44	(Srivastava et al. 2015)
	*Haloferaxalexandrinus* GUSF-1	Cell lysate	Te(IV)	Rod-shaped	40 × 7	(Alvares and Furtado 2021)

**Fig. 4:**
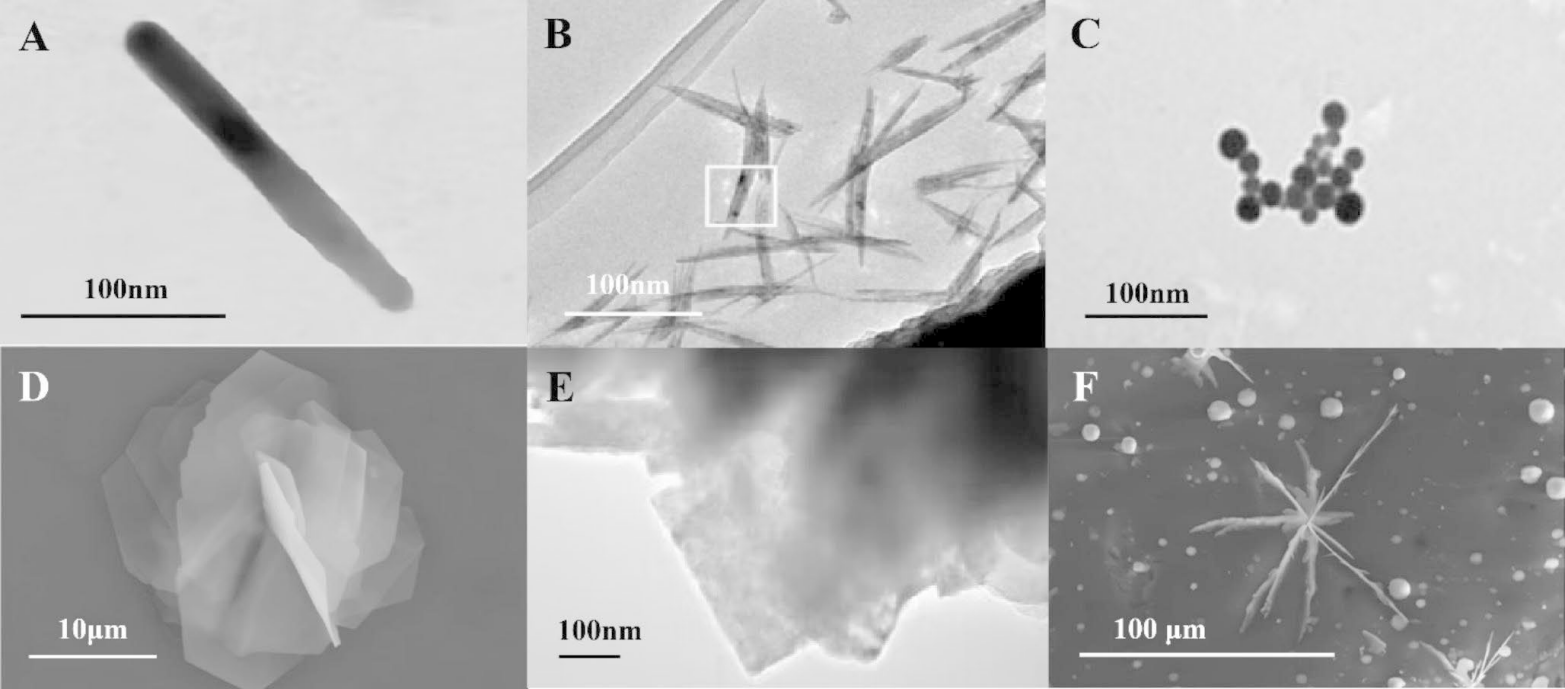
TEM images of different shapes of Bio-TeNPs. (**A**) Rod-like TeNPs (Shakibaie et al. 2017), (**B**) needle-like TeNPs (Borghese et al. 2020), (**C**) spherical TeNPs (Nwoko et al. 2021), and (**D**) nanoflower, (**E**) nanoplate and (**F**) nanobranch (Wang et al. 2018), respectively

Microbial Bio-TeNPs were usually located in the cytoplasm, periplasmic space or outside the cell. Additionally, Bio-TeNPs could accumulate and be excreted from cells after forming inside the cells, which could then exist both inside and outside the cell, such as occurs in the *Raoultella* genus and *Escherichia* genus (Nguyen et al. 2019). The production of Bio-TeNPs involves two steps, reduction and precipitation, occurring either intracellularly or extracellularly, which might be related to the reductase or reducing substances at different sites (Borghese et al. 2017). So far, most microbially sourced Bio-TeNPs are derived from intracellular pathways. In terms of shape, Bio-TeNPs are formed mainly in the shape of rods, needles and spheres, but also nanoflowers, nanoplates, and even nanobranches in a few microbes (Fig. 4). Interestingly, *Rhodococcus aetherivorans* BCP1 converted tellurite to produce spherical tellurium nanoparticles that could further transform into nanorods with increasing exposure time (Presentato et al. 2018). Owing to electrostatic interactions, Bio-TeNPs adhered to each other and formed large rose-shaped knots in *Pseudomonas pseudoalcaligenes* (Shakibaie et al. 2017).

The variety of microorganism species, growth medium and synthesis conditions all have an effect on the size, shape and dispersion of nanoparticles (Barabadi et al. 2018). The optimal metal ion concentration, pH and reaction temperature are also regarded as important factors in the formation of nanoparticles (Marooufpour et al. 2019). The links between reduction kinetics and protein structure may also lead to morphological differences in biological tellurium nanoparticles, according to the study of *Lysinibacillus* sp. ZYM-1 cells (Wang et al. 2018). The type of tellurate reductase presented on the cell membrane may determine the morphology of Bio-TeNPs, including the simultaneous presence of nanorods, nanoflowers, nanobranches, and nanoplates (Wang et al. 2018). The mechanism underlying the different positions and shapes of Bio-TeNPs produced by different microorganisms, as well as the variations of position and shape for Bio-TeNPs produced by the same microorganisms, are still unclear and need further investigation.

### Application of microbial Bio-TeNPs

Nanomaterials have the advantages of larger surface area, higher cytocompatibility and fewer defects than other materials, and they are used in almost all fields, such as biomedicine, optoelectronics and environmental remediation (Zonaro et al. 2015; Vaigankar et al. 2018; Vahidi et al. 2021). Microbial Bio-TeNPs are typically generated under mild reaction conditions. They provide a safe, economical and environmentally friendly means to reduce the use of organic solvents and more toxic reactants in a variety of applications, reducing the production of toxic residues with great prospects for further industrial development (Borghese et al. 2020).

**Fig. 5:**
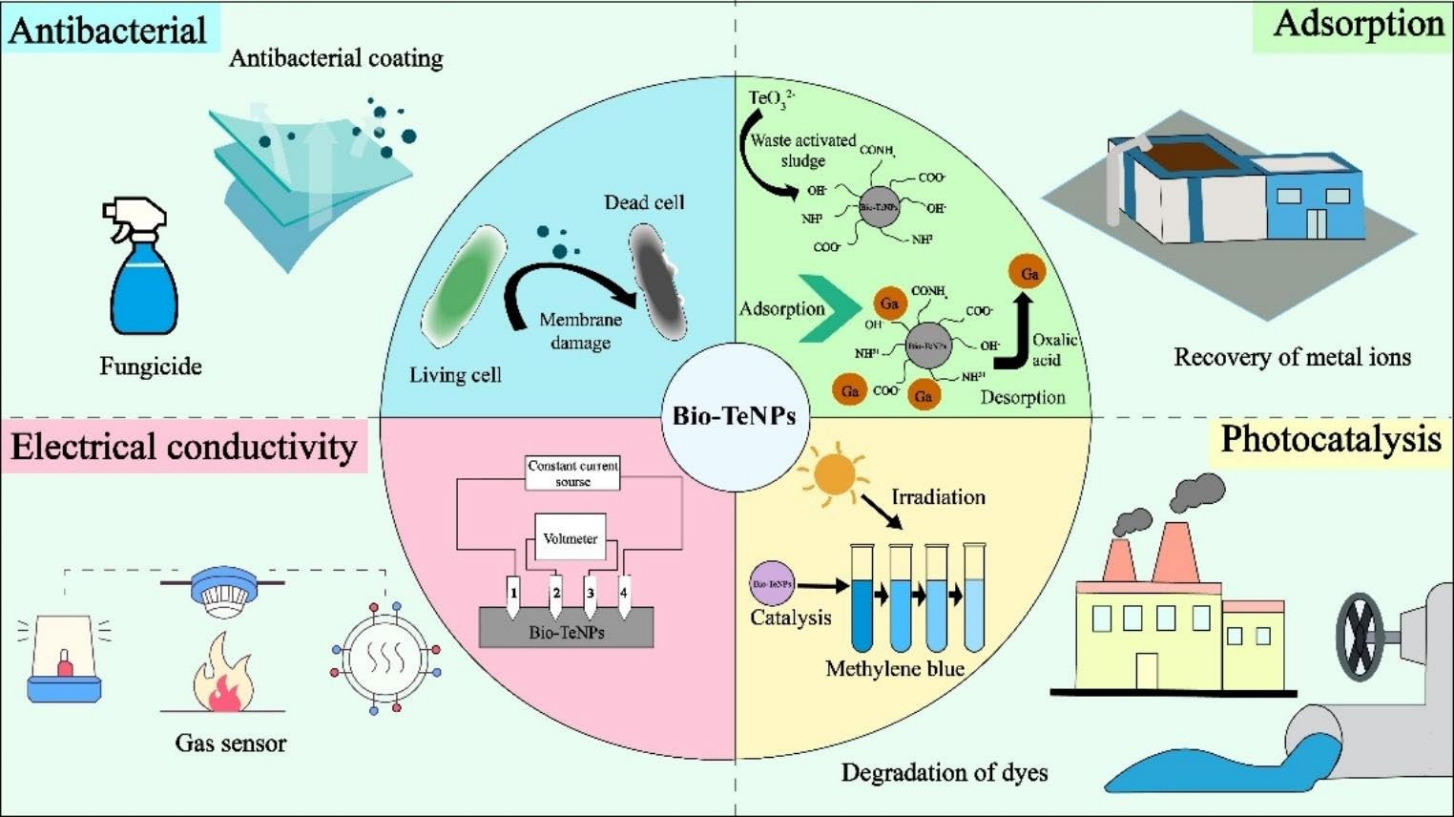
The application of Bio-TeNPs

Microbial Bio-TeNPs show great potential for applications in antibacterial, adsorptive, photocatalytic and conductive electronic materials (Fig. 5). (1) Bio-TeNPs have been found to have excellent antibacterial activity (Table 4). Toxicity of Bio-TeNPs, while less than that of soluble Te oxyanions, is still significant and the Bio-TeNPs are able to destroy biofilms, produce reactive oxygen species, damage DNA and release toxic ions in specific contexts (Ghosh et al. 2021). Thus, Bio-TeNPs were found to inhibit the growth of *E. coli* (Pugin et al. 2014), *Staphylococcus aureus* (Abed et al. 2023), *K*. *pneumoniae*, *Pseudomonas aeruginosa* (Zare et al. 2012), *Candida albicans* (Zare et al. 2014), *Aspergillus flavus* and *Aspergillus niger* (El-Sayyad et al. 2020). Najimi et al. (2017) conducted a subacute evaluation of Bio-TeNPs using mice prepared by *P. pseudoalcaligenes*. They found that the toxicity of Bio-TeNPs was lower than that of tellurite, and in a 14-day subtoxicity study in mice, doses below 1.2 mg/kg did not cause adverse reactions. Furthermore, Abed et al. (2023) tested Bio-TeNPs in a rat intravenous infection model, which demonstrated their effectiveness against methicillin-resistant *S. aureus* (MRSA) and improved the survival rate of infected animals, while also showing a reasonable level of safety in terms of liver and kidney function. Bio-TeNPs are expected to be an alternative to traditional antibiotics and chemical fungicides used in antibacterial coatings of medical devices (Zonaro et al. 2017), alleviating the pressure of microbial antibiotics resistance caused by conventional antibiotics. (2) Bio-TeNPs are also good adsorbents. Bio-TeNPs produced by *Shinella* sp. WSJ-2 were able to remove a variety of dyes and metal ions due to electrostatic interactions (Wu et al. 2019). Bio-TeNPs can recover gallium by adsorption of up to 74 mg of Ga^3+^ per gram (Saikia et al. 2022). (3) Bio-TeNPs also exhibited photocatalytic ability. Bio-TeNPs achieved 90% photocatalytic degradation of the dye methylene blue in *Shewanella baltica* within 4 h. Therefore, they can be used as a photocatalyst for the remediation of methylene blue in industrial wastewater (Vaigankar et al. 2018). (4) Bio-TeNPs with good conductivity synthesized by *R. aetherivorans* BCP1 demonstrate potential for electronic applications (Presentato et al. 2018). They can be used in optoelectric, thermoelectric, piezoelectric devices, as well as gas sensors and infrared detectors. At present, the applications of Bio-TeNPs are mainly focused on antibacterial applications, while less attention has been paid to their toxicity to animals or humans. Additionally, studies on adsorption, photocatalysis and optical properties of Bio-TeNPs are still in their infancy and other applications need to be further studied.

**Table 4:** Antibacterial activity of Bio-TeNPs

Bio-TeNPs source	Pathogens	Ref.
*A. pittii*	*E*. *coli*.	(Tang et al. 2022)
*Bacillus* sp. BZ	*S*. *aureus*, *S.typhi*, *K*. *pneumonia* and *P*. *aeruginosa*	(Zare et al. 2012)
*Bacillus* sp. BZ	*C. albicans* ATCC14053	(Zare et al. 2014)
*H. salifodinae* BK3	*E*. *coli* NCIM2345, *P. aeruginosa* MTCC2581, *S*. *aureus* MTCC737 and *Micrococcus luteus*	(Srivastava et al. 2015)
*Ochrobactrum* sp. MPV1	*E*.*coli* JM109, *P. aeruginosa* PAO1 and *S. aureus* ATCC25923	(Zonaro et al. 2015)
*Pseudomonas* sp. strain BNF22	*E*. *coli*.	(Pugin et al. 2014)
*P. pseudoalcaligenes*	*E*. *coli*, *P. aeruginosa*, *S. typhi*, *S. aureus* (MRSA), *C. albicans*, and *Candida dubliniensis*	(Shakibaie et al. 2017)
*S. cyaneus*	*A. flavus*, *A. niger*, *Aspergillus fumigatus*, *P. aeruginosa*, *S*. *aureus* and *K. pneumoniae*	(El-Sayyad et al. 2020)
*S. graminisoli*	Methicillin-resistant *S*. *aureus* (MRSA)	(Abed et al. 2023)

### Conclusion and perspectives

In recent years, with the increasing demand for tellurium, concerns about environmental pollution and toxicity associated with tellurium have gained widespread attention. Microbes play an important role in the tellurium biogeochemical cycle. Several different mechanisms for tellurium detoxification have been identified in microorganisms. Among the detoxification mechanisms, the conversion of highly toxic tellurate and tellurite oxyanions into lower-toxicity elemental tellurium or methylated organotellurium compounds through microbial processes has been demonstrated as an environmentally friendly, feasible, and promising approach to deal with tellurite and tellurate contamination. Moreover, some microorganisms could reduce tellurite or tellurate to elemental tellurium and form Bio-TeNPs. Bio-TeNPs show great potential applications in fields including biomedicine, optoelectronics and environmental remediation. Future research is expected to focus on the following aspects: (1) tellurium pollution monitoring in the environment, (2) migration and transformation of tellurium in the natural environment, (3) tellurite and tellurate reduction mechanisms under anaerobic conditions, (4) high efficiency tellurite and tellurate reductases, (5) identification of tellurate methylase, (6) Bio-TeNPs formation and secretion mechanism, (7) the toxicity evolution of Bio-TeNPs, (8) more application directions of Bio-TeNPs.

## Data Availability

The data supporting the findings of this review are available in the references cited within this article.
